# Amniotic Fluid Arginine from Gestational Weeks 13 to 15 Is a Predictor of Birth Weight, Length, and Head Circumference

**DOI:** 10.3390/nu9121357

**Published:** 2017-12-14

**Authors:** Astrid Bjørke-Jenssen, Per Magne Ueland, Anne-Lise Bjørke-Monsen

**Affiliations:** 1 Faculty of Medicine and Health Sciences, Norwegian University of Science and Technology, 7491 Trondheim, Norway; astridbjenssen@gmail.com; 2 Laboratory of Clinical Biochemistry, Haukeland University Hospital, 5021 Bergen, Norway; Per.Ueland@k2.uib.no; 3 Institute of Medicine, University of Bergen, 5021 Bergen, Norway

**Keywords:** amino acids, arginine, fetal growth, pregnancy, placentation

## Abstract

Arginine is a constituent of proteins and a precursor for polyamines and nitric oxide, and is essential for placentation, angiogenesis, and growth. Maternal plasma arginine concentrations are found to be lower in pregnancies complicated by fetal growth restriction, and arginine supplementation in later pregnancy is reported to increase birth weight. We measured arginine and the metabolites asymmetric dimethylarginine (ADMA) and symmetric dimethylarginine (SDMA) in the amniotic fluid obtained in pregnancy weeks 13 to 15 from 363 pregnancies with a documented normal outcome and related the concentrations to birth weight, length, and head circumference. Arginine was higher in the amniotic fluid from female (mean 40.8 (SD 10.6) µmol/L) compared to male fetuses (37.4 (SD 11.2) µmol/L, *p* = 0.003). Despite the gender difference, arginine in the amniotic fluid from gestational weeks 13–15 was the strongest predictor for birth weight, length, and head circumference. ADMA was a strong predictor for birth weight and length, SDMA for birth weight, while Arg/ADMA and Arg/SDMA only predicted head circumference in multiple linear regression models. Due to increased arginine demands, pregnancy is considered a state of relative arginine deficiency. Our findings reflect the importance of a good maternal arginine status in early pregnancy, an observation that should be evaluated in an intervention study.

## 1. Introduction

Arginine plays an essential role in fetal and placental development and is reported to be abundant in the amniotic fluid in early pregnancy [1,2,3] Arginine serves as a building block of proteins and a key regulator of several metabolic pathways [4]. Arginine is hydrolyzed to ornithine and converted into the polyamines putrescine, spermine, and spermidine [5], key regulators of placental angiogenesis, trophoblast growth, and embryogenesis [6]. 

Arginine is additionally the substrate for the production of nitrix oxide (NO), and asymmetric dimethylarginine (ADMA), a guanindine (*N^G^*)-dimethylated derivate of arginine, is a strong direct inhibitor of nitric oxide synthase (NOS). Symmetric dimethylarginine (SDMA) is an indirect and weaker inhibitor of NOS and a marker of renal function. The ratio Arg/ADMA is considered to be a marker of NOS activity [7], and a low ratio is regarded as a marker of endothelial dysfunction [8]. Pregnancy is associated with enhanced vasodilatation, and NO has been assigned a particular role in the physiological vascular adaptation [9] that is considered to be essential for the formation of a healthy endothelium and for promoting the endovascular invasion by the cytotrophoblast [10].

Due to the increased needs for arginine for placental and fetal growth [11], pregnancy is considered to be a state of relative arginine deficiency [12]. Maternal plasma arginine concentrations were found to be lower in pregnancies complicated by fetal growth restriction [13,14]. 

Before keratinization of the fetal skin occurs around gestational week 20, the composition of the amniotic fluid is considered to reflect the metabolite profile of fetal plasma and provides a rational compartment for studies of fetal nutrition and metabolism [1].

We have measured arginine and the metabolites ADMA and SDMA in amniotic fluid obtained in pregnancy weeks 13 to 15 from pregnancies with a documented normal outcome. The aim of the study was to evaluate the relation between these amino acids and the final birth weight, length, and head circumference.

## 2. Materials and Methods 

Samples of amniotic fluid taken by transabdominal amniocentesis for genetic prenatal diagnosis at Haukeland University Hospital, Bergen, Norway, during 1995 through 1998 were eligible for this observational study. The gestational age was determined by menstrual dates and checked by ultrasound examination. The information about pregnancy outcomes was collected from questionnaires routinely returned after delivery. Samples from singleton pregnancies with a documented normal outcome, obtained at gestational weeks 13, 14, and 15 were included in the study (*n* = 371). Birth before gestational week 37 was defined as premature birth, and a birth weight below 2500 grams at term was defined as small for gestational age (SGA).

The samples were stripped for identifiers, the procedures for information collection were in accordance with the revised Helsinki declaration of 1983, and the study was granted approval by the Regional Committee on Medical Research Ethics West (365/02).

### 2.1. Sampling and Biochemical Analysis

The samples were centrifuged after amniocentesis and stored at −20 °C until analyses were done in 1999. Amniotic fluid concentrations of arginine, ADMA, and SDMA were measured using a LC-MS/MS method [15] by the laboratory Bevital AS, Bergen Norway (www.bevital.no).

### 2.2. Statistical Analysis

Data are presented as mean and SD and compared by ANOVA test, or as median and interquartile range (IQR) and compared by Kruskal–Wallis test. Multiple linear regression models, corrected for gender, gestational age at sampling, and storage period were used to explore the relationships between amniotic fluid arginine, ADMA, or SDMA concentrations and growth parameters at birth (weight, length, and head circumference). Graphical illustrations of the relationships between arginine or the methylarginines and birth weight were obtained by generalized additive models (GAM), corrected for gender, gestational age at sampling, and storage period.

The SPSS statistical program (version 23) and the package “mgcv” in *R*, version 3.3 (The *R* Foundation for Statistical Computing, Vienna, Austria) were used for the statistical analyses. Two-sided *p*-Values < 0.05 were considered statistically significant. 

## 3. Results

### 3.1. Characteristics of the Study Population

The main indication for amniocentesis was maternal age (Table 1). The infants were healthy and born at term with an appropriate weight for gestational age, apart from three infants (0.8%) who were born premature and six term-born infants (1.6%) who were defined SGA. These nine infants were excluded, thereby leaving amniotic fluid from 363 pregnancies with a documented normal outcome eligible for the study. 

The mean birth weight was higher in boys compared to girls (3713 (SD 562) versus 3592 (SD 468) g, *p* = 0.03), and the same pattern was seen for birth length (50.8 (SD 2.8) versus 49.9 (SD 2.4) cm, *p* = 0.005) and head circumference (35.7 (SD 1.4) versus 35.2 (SD 1.5), cm, *p* = 0.008). 

### 3.2. Arginine and Metabolite Concentrations According to Gestational Age

The mean arginine (−13%), ADMA (−15%), and SDMA (−10%) concentrations decreased significantly from gestational week 13 to 15, while no such trend was seen for the ratios Arg/ADMA and Arg/SDMA (Table 2). The storage time varied from 1 to 4 years, with no difference according to the gestational week at sampling (*p* = 0.41).

### 3.3. Arginine and Metabolite Concentrations According to Gender

The mean arginine concentrations were higher in the amniotic fluid from female (40.8 (SD 10.6) μmol/L) compared to male fetuses (37.4 (SD 11.2) μmol/L, *p* = 0.003), whereas no differences were seen for ADMA (*p* = 0.16) and SDMA (*p* = 0.30) according to gender. 

The mean Arg/ADMA ratio was higher in the amniotic fluid from female (15.3 (SD 3.3)) compared to male fetuses (14.4 (SD 3.5), *p* = 0.007), whereas no significant difference according to gender was seen in the Arg/SDMA ratio (*p* = 0.07). 

### 3.4. Arginine and Metabolite Concentrations as Determinants for Birth Weight, Length, and Head Circumference

Birth weight was positively correlated to arginine (*r* = 0.23, *p* < 0.001) and ADMA (*r* = 0.25, *p* < 0.001), weakly correlated to SDMA (*r* = 0.17, *p* = 0.001), and not correlated to Arg/ADMA (*p* = 0.26) and Arg/SDMA ratios (*p* = 0.21) by Spearman correlation. Positive correlations were also seen for birth length versus arginine (*r* = 0.14, *p* = 0.02), ADMA (*r* = 0.18, *p* = 0.002), and SDMA (*r* = 0.12, *p* = 0.04), but not versus Arg/ADMA (*p* = 0.97) or Arg/SDMA (*p* = 0.66) ratios. Head circumference was positively correlated only to arginine (*r* = 0.17, *p* = 0.01). 

Amniotic fluid arginine was the strongest predictor for birth weight, length, and head circumference in multiple linear regression models which additionally included gender, gestational week, and storage period (Table 3). ADMA was a strong predictor for birth weight and length, SDMA for birth weight, while the ratios Arg/ADMA and Arg/SDMA were significant predictors only for head circumference (Table 3). 

**Figure 1:**
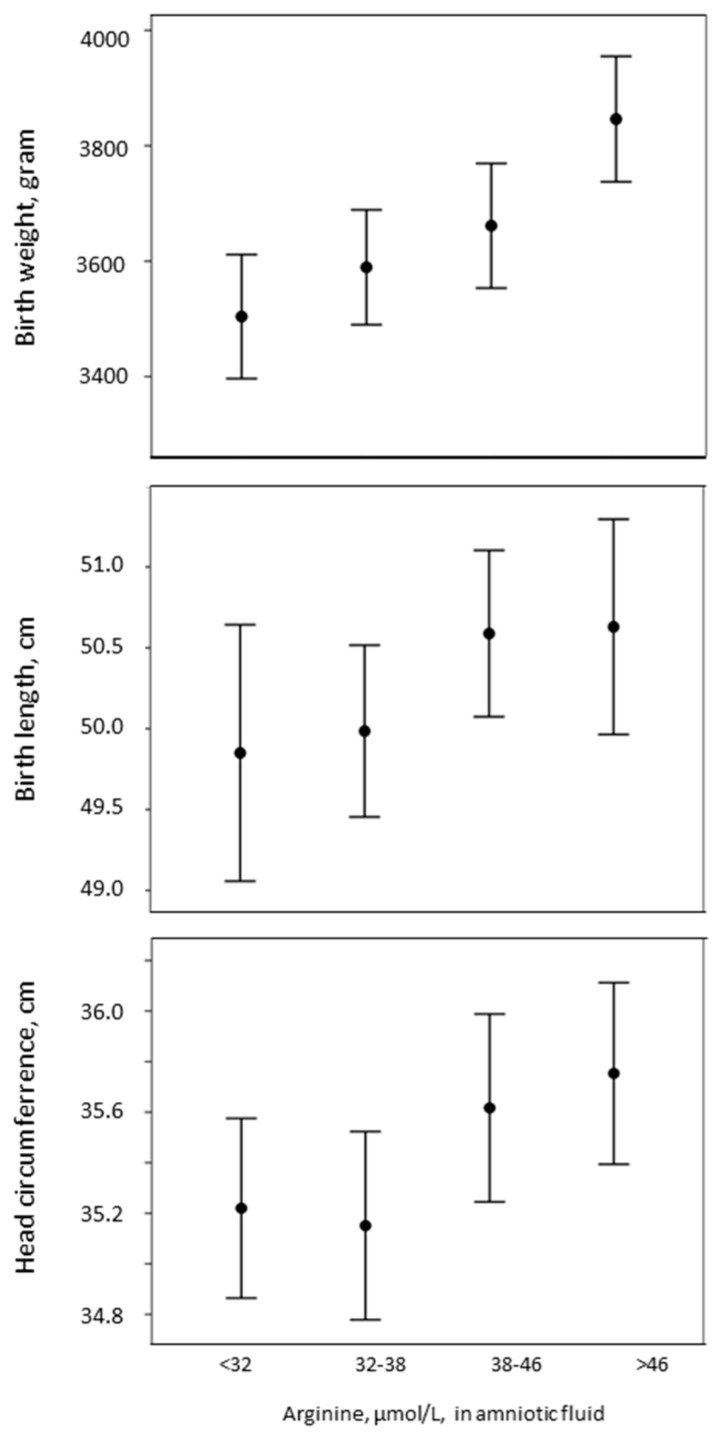
Changes in birth weight, length, and head circumference with respect to amniotic fluid arginine concentrations, in quartiles.

Birth weight (*p* < 0.001), length (*p* = 0.19), and head circumference (*p* = 0.05) increased with increasing arginine concentrations in the amniotic fluid (Figure 1).

**Figure 2:**
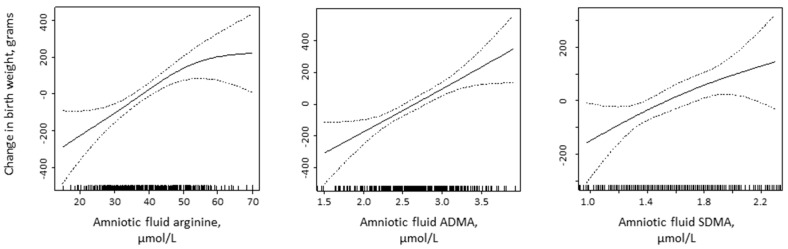
Changes in birth weight in relation to arginine, ADMA, and SDMA concentrations in the amniotic fluid from gestational weeks 13–15 by generalized additive models (GAM).

A dose–response relationship between birth weight and arginine, ADMA, and SDMA was visualized by GAM curves corrected for gender, gestational week, and storage time (Figure 2).

## 4. Discussion

Arginine, ADMA, and SDMA decreased in the amniotic fluid from gestational weeks 13–15. Arginine was higher in the amniotic fluid from female compared to male fetuses. Arginine was the strongest predictor for birth weight, length, and head circumference, followed by ADMA for birth weight and length and SDMA for birth weight, while the ratios Arg/ADMA and Arg/SDMA were only related to head circumference. 

### 4.1. Strength and Limitations

The amniotic fluid samples were taken for routine prenatal diagnostic purposes and stored at a nonoptimal temperature (minus 20 °C) for 1–4 years before analysis, which may influence arginine and metabolite concentrations. However, under these conditions, ADMA and SDMA are stable in both serum and plasma, whereas arginine may increase about 2.5% per year in serum (but not in plasma), a change that has been attributed to proteolysis [16]. However, there was no difference in the storage time in relation to the gestational week at sampling. 

As this study was done on anonymous samples, the information on the maternal status was limited, and factors known to be related to birth outcome, like prepregnancy body mass index, parity, smoking, and socioeconomic status, were not available [17]. However, all samples included in the study had a documented normal outcome.

### 4.2. Gender Differences

We found higher concentrations of arginine, no difference in ADMA and SDMA, and a higher Arg/ADMA ratio in the amniotic fluid in pregnancies from female compared to male fetuses. The same gender difference, with higher plasma arginine concentrations in females, was observed in mice [18]. 

Amniotic fluid testosterone concentration is reported to be higher in male than female fetuses at gestational week 15 [19]. As testosterone is known to stimulate arginase [20], this may contribute to the observed lower arginine concentrations observed in the amniotic fluid from male fetuses [18]. 

### 4.3. Amniotic Fluid Arginine Levels during Pregnancy

Published data on amniotic fluid arginine concentrations are scarce, and data on ADMA and SDMA are missing. We found that arginine concentrations decreased from gestational week 13 to 15, and the mean concentration obtained in week 15 (36.0 (SD 8.3) µmol/L) resembles published data from one study on amniotic fluid from pregnancy week 15, mean 34.8 (SD 12) µmol/L [21]. 

### 4.4. Arginine, ADMA, and SDMA in the Amniotic Fluid Compared to Maternal Plasma and Cord Blood

Amniotic fluid volume increases from about 25 mL at 10 weeks to about 400 mL at 20 weeks. Before keratinization of the fetal skin, amino acids and other nutrients diffuse into the amniotic fluid from the placenta through the placental membranes, and from the fetal circulation through the fetal skin, and the composition of the amniotic fluid is considered to reflect that of fetal plasma [1]. 

However, the concentrations of arginine, ADMA, and SDMA that we observed in the amniotic fluid differed substantially from the published concentrations in maternal plasma in early pregnancy and in cord blood at term. The mean amniotic fluid arginine concentration at week 15 (36.0 (SD 8.3) µmol/L) was less than half (42%) of the one that was reported in maternal plasma at pregnancy week 16 (estimated mean 85.5 (SE 4.6) µmol/L) [22], and in the umbilical cord vein at term (median 85.3 (IQR 69.7–95.0) µmol/L [22,23]. In pregnancy, there is a high demand for arginine for the production of polyamines and NO [4], so the lower arginine levels in the amniotic fluid may reflect arginine consumption by the placenta and the fetus. The placenta lacks arginase, a strategy which is suggested to maximize the availability of arginine in the systemic circulation from mother to fetus [4].

The mean ADMA concentration in the amniotic fluid at week 15 (2.28 (SD 0.38) µmol/L) was substantially higher (585%) compared to maternal plasma ADMA concentrations at pregnancy week 16 (estimated mean 0.39 (SE 0.02) µmol/L) [22], and substantially higher (178%) compared to the umbilical cord vein at term (median 1.28 (IQR 1.18–1.38) µmol/L) [23]. Our mean SDMA in the amniotic fluid at week 15 (1.41 (SD 0.31) µmol/L) was also higher (353%) than in maternal plasma (estimated mean 0.40 (SE 0.02) µmol/L) [22], but slightly lower (83%) than in the umbilical cord vein at term (median 1.69 (IQR 1.48–2.01) µmol/L) [23]. 

It has been suggested that the placenta is the primary source of ADMA present in fetal circulation and that the observed high ADMA concentration may be a compensatory mechanism to stabilize the high NO concentrations in the fetoplacental unit [23]. 

The amniotic fluid Arg/ADMA ratio at week 15 (16.1 (SD 4.0) was only 7% of reported maternal plasma ratio in pregnancy week 16 (estimated mean 226.8, (SE 12.4)) [22], and 24% of reported median umbilical cord vein ratio at term (66.0 (IQR 56.3–72.9) [23]. 

### 4.5. Arginine, ADMA, and SDMA as Determinants for Fetal Growth

Arginine was the strongest predictor for birth weight, length, and head circumference, followed by gender. The metabolites ADMA and SDMA were weakly related to birth weight and length, while the ratios Arg/ADMA and Arg/SDMA were weakly related to head circumference. 

Published data on the relation between birth weight and amniotic fluid concentrations of arginine, ADMA, and SDMA are missing. No significant correlations between circulating arginine, ADMA, SDMA levels, and birth weight were seen in 130 neonates born premature (<30 gestational week) with a very low birth weight (<1500 grams) [24]. However, maternal plasma arginine concentrations were found to be lower in pregnancies complicated by fetal growth restriction [13,14].

Our findings reflect the importance of arginine in pregnancy, derived from its diverse functions, both as a constituent of proteins and as a precursor for polyamines and NO, which make it essential for growth and angiogenesis. An adequate concentration of arginine was shown to be crucial for placentation, utero–placental blood flow and transfer of nutrients to the fetus, embryogenesis, and fetal growth and development [25]. Arginine concentrations markedly increase in the uterine lumen during the peri-implantation period of pregnancy [26,27], and arginine is also abundant in the conceptus [28]. The placental synthesis of both NO and polyamines in animal studies was found to be highest during early gestation when placental growth is most rapid [5,29,30]. Whether this is the case for humans is unknown. 

Both animal and human studies have shown enhanced placental–fetal development and growth after arginine supplementation in pregnancy [31,32,33]. The human studies were done in pregnancies with a diagnosis of gestational hypertension [31] or intrauterine growth retardation [32,33]. The supplementation was initiated in middle or late pregnancy and was associated with increased birth weight [32,33], reductions in diastolic blood pressure, and prolonged pregnancy [31].

## 5. Conclusions

Arginine in the amniotic fluid from gestational weeks 13 to 15 was found to be a strong predictor of birth weight, length, and head circumference, while ADMA was a predictor for birth weight and length and SDMA only for birth weight. 

Our findings reflect the importance of arginine metabolism in pregnancy, because of its diverse functions, both as a constituent of proteins and as a precursor for polyamines and NO, which make it essential for placentation, angiogenesis, and growth. Whether an improved maternal arginine status in early pregnancy may enhance fetal growth, should be evaluated in an intervention study.

## Figures and Tables

**Table 1:** Characteristics of the population (*n* = 363).

Indications for Amniocentesis	
Maternal age > 38 years, *n* (%)	315 (87)
Previous child with chromosomal aberration or malformation, *n* (%)	31 (8)
Maternal epilepsy, *n* (%)	6 (2)
Other, *n* (%)	11 (3)
Maternal age, years, median (total range)	39.0 (24.0–45.0)
Gestational age, week, median (total range)	14.0 (13.0–15.0)
Male sex, *n* (%)	180 (50)
Birth weight, g, mean (SD)	3650 (520)
Birth length, cm, mean (SD)	50.3 (2.6)
Head circumference, cm, mean (SD)	35.5 (1.4)
Apgar score 1, mean (SD)	9 (1)
Apgar score 2, mean (SD)	9 (1)

**Table 2:** Concentrations of arginine, asymmetric dimethylarginine (ADMA), and symmetric dimethylarginine (SDMA) in amniotic fluid (*n* = 363) according to the pregnancy week.

Parameters	Pregnancy Week			*p*-Value
	13 (*n* = 110)	14 (*n* = 217)	15 (*n* = 36)	
Sampling year	1996 (1995–1997)	1996 (1995–1997)	1996 (1995–1996)	0.26 *
Arginine, μmol/L	41.3 (11.4)	38.4 (11.1)	36.0 (8.3)	0.019 **
Asymmetric dimethylarginine, μmol/L	2.71 (0.52)	2.66 (0.49)	2.28 (0.38)	<0.001 **
Symmetric dimethylarginine, μmol/L	1.59 (0.34)	1.59 (0.33)	1.41 (0.31)	0.008 **
Arg/ADMA	15.2 (3.3)	14.5 (3.4)	16.1 (4.0)	0.015 **
Arg/SDMA	26.6 (7.7)	24.7 (7.0)	26.6 (7.8)	0.041 **

* The values are given as median (IQR) and compared by the Kruskal–Wallis test. ** The values are given as mean (SD) and compared by ANOVA.

**Table 3:** Arginine and metabolites in the amniotic fluid as determinants of birth weight, length, and head circumference by multiple linear regression.

Independent Variables *	Dependent Variables								
	Birth Weight (g) (*n* = 363)			Birth Length (cm) (*n* = 275)			Head Circumference (cm) (*n* = 239)		
	B	95% CI	*p*-Value	B	95% CI	*p*-Value	B	95% CI	*p*-Value
Arginine	116	(69, 174)	<0.001	0.4	(0.1, 0.7)	0.01	0.3	(0.1, 0.4)	0.003
ADMA	99	(50, 147)	<0.001	0.3	(0.1, 0.6)	0.02	0.2	(−0.03, 0.3)	0.094
SDMA	68	(19, 116)	0.006	0.3	(0.0, 0.6)	0.06	0.1	(−0.1, 0.3)	0.24
Arg/ADMA	30	(−18, 79)	0.22	0.04	(−0.2, 0.3)	0.79	0.2	(0.0, 0.3)	0.05
Arg/SDMA	57	(9, 105)	0.02	0.07	(−0.2, 0.4)	0.64	0.2	(0.01, 0.3)	0.03

* Separate models were used for each amino acid and for the ratios. Each model additionally contained gender, gestational age, and storage period. Amino acids and ratios were categorized as quartiles.
